# Native lagomorphs suppress grass establishment in a shrub‐encroached, semiarid grassland

**DOI:** 10.1002/ece3.4730

**Published:** 2018-12-18

**Authors:** Samuel T. Abercrombie, John L. Koprowski, Mary H. Nichols, Jeffrey S. Fehmi

**Affiliations:** ^1^ School of Natural Resources and the Environment University of Arizona Tucson Arizona; ^2^ USDA‐ARS Southwest Watershed Research Tucson Arizona

**Keywords:** Arizona, *Eragrostis lehmanniana*, herbivore exclosures, herbivory, shrub encroachment, *Sylvilagus*

## Abstract

Shrub encroachment into arid grasslands has been associated with reduced grass abundance, increased soil erosion, and local declines in biodiversity. Livestock overgrazing and the associated reduction of fine fuels has been a primary driver of shrub encroachment in the southwestern United States, but shrublands continue to persist despite livestock removal and grassland restoration efforts. We hypothesized that an herbivory feedback from native mammals may contribute to continued suppression of grasses after the removal of livestock. Our herbivore exclusion experiment in southeastern Arizona included five treatment levels and allowed access to native mammals based on their relative body size, separating the effects of rodents, lagomorphs, and mule deer. We included two control treatments and replicated each treatment 10 times (*n = *50). We introduced uniform divisions of lawn sod (*Cynodon dactylon*) into each exclosure for 24‐hr periods prior to (*n = *2) and following (*n = *2) the monsoon rains and used motion‐activated cameras to document herbivore visitations. In the pre‐monsoon trials, treatments that allowed lagomorph access had less sod biomass relative to other treatments (*p < *0.001), averaging 44% (*SD* 36%) and 29% (*SD* 45%) remaining biomass after the 24‐hr trial periods. Following the onset of monsoons, differences in remaining biomass among treatments disappeared. Desert cottontails (*Sylvilagus audubonii*) were detected more frequently than any of the other 11 herbivore species present at the site, accounting for 83% of detections during the pre‐monsoon trials. Significantly more (*p < *0.001) desert cottontails were detected during the pre‐monsoon trials (2,077) compared to the post‐monsoon trials (174), which coincided with biomass removal from lagomorph accessible treatments. We conclude that desert cottontails are significant consumers of herbaceous vegetation in shrub‐encroached arid grasslands and they, along with other native herbivores, may act as a biotic feedback contributing to the competitive advantage and persistence of shrubs.

## 1 INTRODUCTION

Worldwide, the dynamics of arid and semiarid grasslands have shifted over the past 150 years to favor the spread and dominance of shrubs over forbs and grasses (Van Auken, 2009). As shrub cover has increased, grass and herbaceous cover has decreased (Archer et al., 2011; Van Auken, 2009), which in turn has commonly led to decreases in local biodiversity (Blaum, Rossmanith, Popp, & Jeltsch, 2007; Ratajczak, Nippert, & Collins, 2012) and increased soil erosion (Ritchie, Nearing, Nichols, & Ritchie, 2005; Schlesinger, Abrahams, Parsons, & Wainwright, 1999). The shift to shrublands can also diminish the economic value of the land, especially for livestock grazing (D'Odorico, Okin, & Bestelmeyer, 2012; Scholes & Archer, 1997). This loss of value, both ecological and economic, has created sustained interest in preventing further shrub encroachment and reverting current shrublands back to grasslands (Archer et al., 2011; Archer & Predick, 2014), but the mechanisms behind shrub dominance remain unclear.

Hypotheses to explain the mechanisms driving the shift from grass to shrub dominance have included changes in soil nutrient availability due to overgrazing (Schlesinger et al., 1990), increased levels of atmospheric CO_2_ (Archer, Schimel, & Holland, 1995), altered disturbance regimes (Fuhlendorf, Archer, Smeins, Engle, & Taylor Jr, 2008), and long‐term changes in climatic conditions (D'Odorico et al., 2012). In the southwestern United States, a common hypothesis is that the reduction in fine fuels by livestock led to a decrease in fire frequency and intensity, which enabled shrubs to establish in areas where they were previously suppressed by recurring, naturally ignited fires (Levi & Bestelmeyer, 2016). Despite its likely role in causing initial shrub invasion, the elimination of livestock (cattle) grazing has not been an effective strategy for reverting shrub‐encroached areas back to their former grassland states (Valone, Meyer, Brown, & Chew, 2002).

Other biotic and abiotic feedbacks aside from grazing such as fossorial activity, soil erosion, and soil nutrient heterogeneity may actively reinforce shrub persistence long after initial encroachment has taken place (D'Odorico et al., 2012; Schlesinger et al., 1990; Whitford, 1993). One source of biotic feedbacks may be herbivory from small mammals, especially rodents and lagomorphs, which suppresses grass reestablishment in shrub‐invaded grasslands and influences vegetation structure and plant assemblages (Heske, Brown, & Guo, 1993; Roth, Whitford, & Steinberger, 2009). In addition to suppressing grass establishment, lagomorphs can consume substantive amounts of grass biomass and otherwise change spatial patterns of herbaceous growth (Marko, Onodi, Kertesz, & Altbacker, 2011; Ranglack, Durham, & Toit, 2015). As the herbaceous plant cover between shrubs decreases and shrub densities increase, the forage conditions can become more favorable for small mammals due to reduced predation risk (Thompson, 1982). Reduced predation risk allows herbivores to forage more freely, increasing herbivory pressure on grasses, forbs, and seeds (Danielet al., 1993a, 1993b; Kerley & Whitford, 2009) and can lead to the potential facilitation of shrub encroachment (Bestelmeyer, Khalil, & Peters, 2007).

The potential of herbivores at a site to reinforce shrub persistence depends on the constituent herbivores’ dietary preference, behavior, and caloric needs. The impact of herbivory on vegetation seems to be well correlated with herbivore body size, as larger herbivores tend to be less selective foragers, while smaller herbivores tend to have more selective dietary preferences and thus a greater impact on plant assemblages (Bakker, Ritchie, Olff, Milchunas, & Knops, 2006; Olofsson, Hulme, Oksanen, & Suominen, 2004). Similarly, herbivore diets vary predictably within and among guilds based on similarities in body size, which constrain digestive capacity and energy requirements (Belovsky, 1986). Separating the effects of herbivores among small, medium, and large size classes may effectively describe the type and amount of vegetation consumed and which animals are responsible for the consumption (Olofsson et al., 2004; Rebollo, Milchunas, Stapp, Augustine, & Derner, 2013).

At a study site in southeastern Arizona, the cessation of livestock grazing more than 50 years ago and a subsequent shrub removal and grass seeding effort more than 30 years ago have not returned the area to a grassland state. Because grass occurs within long‐term herbivore exclosures, but remains generally absent anywhere else on the site, grass establishment may be influenced by native herbivores. Supporting this idea, Woolhiser, Goodrich, Emmerich, and Keefer (1990) anecdotally attributed the lack of herbaceous vegetation on this site at least in part to rabbits grazing heavily on the grasses that germinated following seeding efforts. We hypothesized that native mammalian herbivores may be suppressing grass establishment and recruitment in the shrub‐dominated former grassland. We expected that the degree of herbivory would vary by herbivore guild due to differences in dietary preferences, metabolic demands, and relative abundance, and that those effects could be differentiated among exclosure treatments based on relative body size. Based on their high detection frequency in pilot studies, we expected that mule deer (*Odocoileus hemionus*) and lagomorphs (desert cottontails [*Sylvilagus audubonii*] and black‐tailed jackrabbits [*Lepus californicus*]) would be the primary consumers of herbaceous vegetation. We also anticipated that the seasonality of forage availability would influence the degree of herbivore pressure, increasing forage demand in the dry period before the primary growing season.

## 2 MATERIALS AND METHODS

### 2.1 Study area

**Figure 1:**
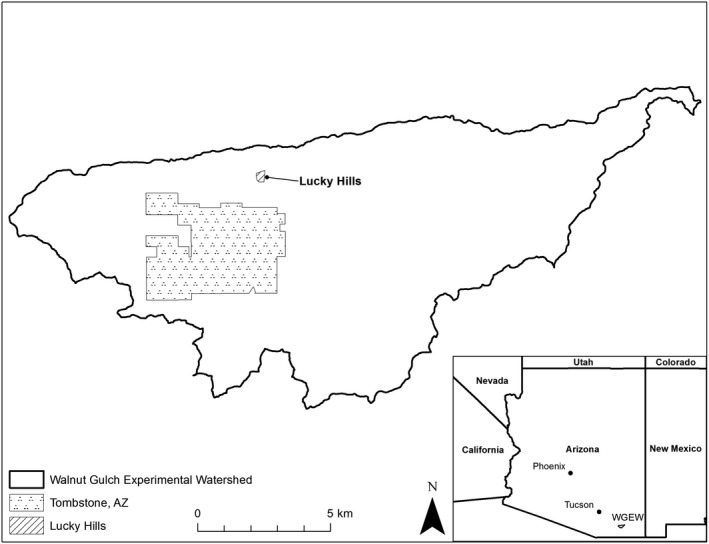
Location of our study site, Lucky Hills (cross‐hatched). The site is a sub‐watershed of the Walnut Gulch Experimental Watershed, which is managed by the USDA and located near the town of Tombstone, Arizona, USA (black dots). The site occurs at 1,370 m elevation, and shrubs cover approximately 20% of the site. Dominant shrub species include whitethorn acacia (*Acacia constricta*), creosote (*Larrea tridentata*), mariola (*Parthenium incanum*), woody crinklemat (*Tiquilia canensis*), and desert zinnia (*Zinnia acerosa*)

Our study site was a 10.92‐ha semiarid sub‐watershed of the Walnut Gulch Experimental Watershed called Lucky Hills (31°44′30″N, 110°3′17″W), located near the town of Tombstone, in Cochise County, Arizona, USA (Figure 1; USDA, 2018). The site was at 1,370 m elevation with a mean annual temperature of 17.6°C and mean annual rainfall of 320 mm since site measurements began in 1953 (USDA‐ARS, 2018). Precipitation primarily occurred during two distinct seasons: the winter (November–March), which provides 30% of annual rainfall, and the summer monsoons (July–September), which provides 50% of annual rainfall (Sheppard, Comrie, Packin, Angersbach, & Hughes, 2002).

The study site was shrub dominated and had a minimal herbaceous layer. The land surface was approximately 55% bare ground, and shrubs covered approximately 20% of the site. Dominant shrub species at the time of this study included whitethorn acacia (*Acacia constricta*), creosote (*Larrea tridentata*), mariola (*Parthenium incanum*), woody crinklemat (*Tiquilia canensis*), and desert zinnia (*Zinnia acerosa*). Lehmann lovegrass (*Eragrostis lehmanniana*) grew abundantly inside of long‐term herbivore exclosures established in the 1980s but was not observed elsewhere. Flushes of forbs and grasses emerged after summer rains, which suggested a viable seed bank remained at the site.

Although livestock grazing had not occurred on the site since 1963, the site was likely severely overgrazed in the late 1800s and early 1900s (Renard, Nichols, Woolhiser, & Osborn, 2008). Due to lack of grass recovery after livestock removal, a chemically based shrub reduction experiment was conducted in the early 1980s with the goal of increasing grass cover, but it was not successful (Emmerich, Helmer, Renard, & Lane, 1984; Ritchie et al., 2005; Woolhiser et al., 1990) and shrub densities had returned to pre‐treatment levels by 2016 (J.R. Smith, *personal communication*, January 11, 2016).

### 2.2 Data collection

Exclosure locations were sited ≥10 m apart using an ArcGIS 10.1 (ESRI, 2012) randomization that excluded roadbeds, washes, gullies, and installed equipment (e.g., rain gauges). Plot locations occurred across a uniform soil type and were largely located on bare ground. If our randomly selected plot locations occurred on top of a large shrub, we moved the point ±1 m so that the vegetation did not exceed ~10 cm in height inside of the exclosure. Locations were then randomly assigned one of five treatment levels, which referred to the size class of mammalian herbivore permitted inside of the exclosure and included the following: (a) SMALL (i.e., rodents), (b) MEDIUM (i.e., rodents plus lagomorphs), (c) LARGE (i.e., mule deer), (d) TOTAL EXCLOSURE (i.e., all mammals prohibited from entry), and (e) TOTAL ACCESS (i.e., all mammals permitted entry). Each treatment level was replicated 10 times. All 50 herbivore exclosures were installed in June of 2016, prior to the start of the monsoon rains.

**Figure 2:**
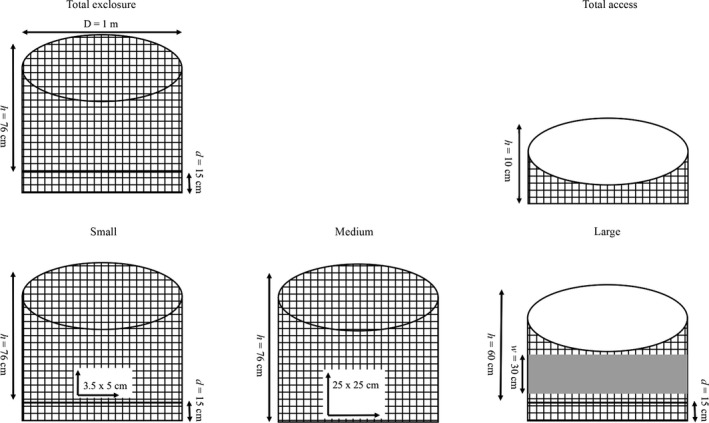
A diagram depicting the construction of each treatment type (not to scale). Each treatment was constructed with the same fundamental design (1‐m‐diameter circular plot) and materials (galvanized steel wire mesh and rebar stakes). We manipulated access points and exclosure heights to achieve five treatment levels. This figure is paired with Table 1, which provides additional information regarding the treatment designs and access manipulations. Exclosure dimensions are provided as following: D = diameter, h = height, d = depth, w = width

We constructed all of the treatments with the same fundamental design and materials and manipulated access points and exclosure heights to achieve the five treatment levels (Table 1, Figure 2). We wrapped galvanized steel wire mesh (6.35‐mm openings) around four rebar stakes to form 1‐m‐diameter circular plots. For the TOTAL EXCLOSURE treatments, we buried the mesh 15 cm below the soil and allowed the mesh to extend 76 cm above the soil surface and covered each top. LARGE treatments were similar to the TOTAL EXCLOSURE treatments, but the mesh height was limited to 60 cm above the ground surface, the tops remained uncovered, and linoleum 30 cm high was wrapped around the outside of the exclosures allowing mule deer to graze while preventing entry by small mammals (e.g., Devall, Parresol, & Smith, 2001). MEDIUM treatments were constructed without burying the mesh, had a mesh top, and 25 × 25 cm openings were cut into opposite sides of the exclosures at ground level. We originally constructed the SMALL treatments without burying the mesh and included 5 × 5 cm openings at ground level. However, we modified this original design after we discovered that desert cottontails had gained access to these exclosures during our first two sod trials, causing us to omit data from the SMALL treatments from our June (eight of 10 exclosures omitted from analysis) and early July (five of 10 exclosures omitted from analysis) trials. We altered the SMALL treatments prior to our second two sod trials (late July and August trials) by burying the mesh to a depth of 15 cm and cutting 3.5 × 5 cm openings on opposite sides of the exclosures at ground level. Finally, we constructed the TOTAL ACCESS treatments by wrapping a strip of mesh 10 cm in height around the rebar stakes and allowed the top of the exclosures to remain open.

**Table 1:** Summary of the design and access manipulations for each exclosure treatment level. Exclosures were constructed with the same basic design and materials, but exclosure heights and opening sizes were manipulated to achieve five treatment levels, which refer to the size class of mammalian herbivore permitted entry: (a) TOTAL ACCESS (i.e., all mammals permitted entry), (b) SMALL (i.e., rodents), (c) MEDIUM (i.e., rodents plus lagomorphs), (d) LARGE (i.e., mule deer), and (e) TOTAL EXCLOSURE (i.e., all mammals prohibited from entry)

Treatment	Herbivores permitted entry	Herbivores denied entry	Depth buried (cm)	Height extended above ground (cm)	Exclosure opening	Exclosure specific variation
TOTAL ACCESS	All	None	n/a	10	Total	
SMALL	Rodents	Lagomorphs, Deer	n/a	~76	3.5 × 5 cm	
MEDIUM	Rodents, Lagomorphs	Deer	15	~76	25 × 25 cm	
LARGE	Deer	Rodents, Lagomorphs	15	60	Open at top	Laminate wrap (30 cm wide)
TOTAL EXCLOSURE	None	All	15	~76	None	

**Figure 3:**
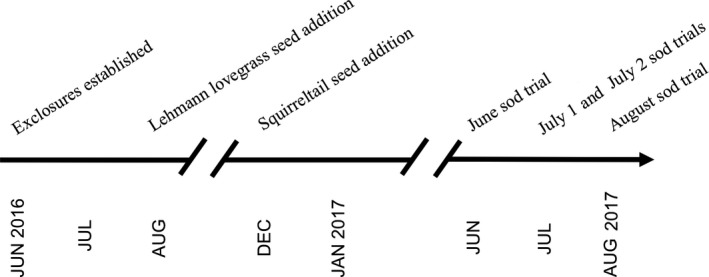
A project timeline highlighting pivotal junctures of the project, including the establishment of the exclosures and the timing of the seed additions and sod trials (note the broken axis). The project took place between 2016 and 2017

We assessed the effects of herbivore exclusion on grass establishment in two ways: seed additions and sod trials. For the seed addition trials, we applied 0.1 g of Lehmann lovegrass seed (Curtis & Curtis, Inc., Clovis, NM, USA) to each exclosure in early August 2016, to coincide with the monsoon rains, and later applied 9.0 g of squirreltail (*Elymus elymoides*: WesternWonder.com, Clovis, NM, USA) seed in mid‐December 2016, to coincide with the winter rains (Figure 3). Total percent grass cover inside each exclosure was visually estimated based on photographs taken inside the exclosures in both March and August of 2017. For the sod trails, we introduced 7.6 × 10.2 cm divisions of fresh lawn sod (Bermuda grass, *Cynodon dactylon*) into each exclosure for two 24‐hr trial periods prior to the onset of monsoon rains (June and early July) and two trial periods following the onset of monsoon rains (late July and early August) of 2017. Following each sod trial, the sod divisions were removed and remaining aboveground biomass was clipped, dried at 70°C for at least 48 hr, and weighed.

To document herbivore activity, we placed a motion‐activated trail camera (Trophy Cam HD Essential, model #119736c; Bushnell Inc., Overland Park, KS, USA) at each exclosure. Cameras were pointed due north with the associated exclosure established squarely in its field of view, and surrounding vegetation was trimmed as needed to reduce misfires. We separated the mammalian herbivores at the site into two dietary functional groups: herbivores and granivores. Herbivores included mule deer, desert cottontails, black‐tailed jackrabbits, and white‐throated woodrats (*Neotoma albigula*). Granivores included kangaroo rats (*Dipodomys merriami*), rock squirrels (*Otospermophilus variegatus*), and “unidentified small rodent,” a category reserved for small rodents that could not be reliably assigned to species via photograph. Because our cameras did not reliably detect animals smaller than white‐throated woodrats, we tallied detections for animals above and below the white‐throated woodrat body size threshold in two different ways. For animals the size of white‐throated woodrats and larger, we only tallied detections of animals physically inside of the exclosures during each sod trial. For animals the size of white‐throated woodrats and smaller (i.e., kangaroo rats and the “unidentified small rodent” detection category), we tallied all detections (inside and around the exclosures) during each sod trial in order to increase our sample size for those species.

We conducted live‐trapping in eight sampling periods between July 2016 and July 2017 to characterize rodent species richness (lagomorphs and mule deer were well characterized from the camera data). We used a 50 × 40 m trapping grid with 25 trap locations spaced at 10 m intervals for each trapping event. At each trap location, we set two sizes (SFA, small; LFA, large) of aluminum folding Sherman Traps (Sherman, Inc., Tallahassee, FL, USA), baited with peanut butter and rolled oats and supplied with cotton for bedding. Traps were set at dusk and checked before sunrise the next morning. All animal handling was in accordance with the Arizona Department of Fish and Game Scientific Collecting License #SP501610 and under the University of Arizona IACUC protocol #16‐169.

### 2.3 Statistical analysis

To analyze the effect of exclosure type and temporal period (month for seed addition trials, trial period for sod trials), a mixed‐model repeated‐measures analysis of variance (ANOVA) was used with the exclosure location as a random variable to analyze the effect of exclosure type and temporal period for both the seed addition and sod trials. For the seed addition trials, we conducted an automated Tukey's Ladder of Powers in R, which provided a lambda value to best approximate a normal distribution (Mangiafico, 2016). To standardize the amount of biomass removed from each division of sod, the weight of the remaining biomass in each individual exclosure was divided by the average weight of remaining biomass from the TOTAL EXCLOSURE treatments for that trial. For documenting herbivores, each photograph of an animal was treated as a single detection. Species detections for each sod trial were tallied, and the raw values are reported. All analyses were conducted in R version 3.3.2 (R Core Team, 2016).

## 3 RESULTS

For the seed addition trials, the overall amount of grass cover produced in the exclosures was extremely low in March ($\overset{¯}{x}$ = 0.61%, *SD* = 0.79%), and although average grass cover increased by an order of magnitude in August ($\overset{¯}{x}$ = 1.6%, *SD* = 2.3%), average cover still remained extremely low. The majority of exclosures had <1% cover (42 in March, 35 in August), and the maximum amount of grass cover measured in an exclosure in either month was 18%.

Significant differences in grass cover among treatment types were driven by reduced cover in treatments accessible to small‐ and medium‐sized herbivores in both the March and the August sampling periods (*F*_4,45_ = 15.18, *p < *0.001). While differences in cover between months were not significant, a significant interaction existed between treatment and sample period (*F*_4,45_ = 3.29, *p = *0.019). In March, the TOTAL ACCESS and SMALL treatments had less overall cover (0.08% for both) relative the TOTAL EXCLOSURE treatments, which had the most overall cover of any exclosure type (2.08%) (Table 2). In August, the TOTAL ACCESS and MEDIUM treatments had the least amount of cover (0.05% and 0.06%, respectively) and both differed significantly from the TOTAL EXCLOSURE treatments, which had an average of 2.74% cover, but the LARGE treatments had the most average cover in this treatment period (3.9%).

**Table 2:** Summary of seed addition results from March and August 2017. Cover estimates were made using photographs of each exclosure during the winter (March) and summer (August) rainy seasons in 2017. Grouping is based on Tukey's HSD post hoc test following the repeated‐measures ANOVA used to test the effect of treatment type on percent grass cover

Exclosure type	Average cover (%)	*SE* (%)	*N*	Group (Tukey)
MARCH				
TOTAL ACCESS	0.08	0.02	10	b
SMALL	0.08	0.033	10	b
MEDIUM	0.14	0.027	10	ab
LARGE	0.67	0.265	10	ab
TOTAL EXCLOSURE	2.08	0.912	10	a
AUGUST				
TOTAL ACCESS	0.05	0.031	10	c
SMALL	1.08	0.991	10	bc
MEDIUM	0.06	0.022	10	c
LARGE	3.9	2.13	10	ab
TOTAL EXCLOSURE	2.74	0.432	10	a

**Figure 4:**
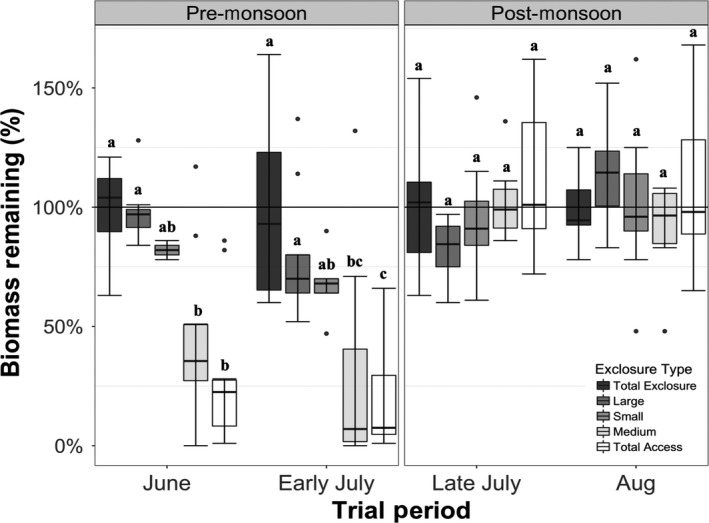
Percent of remaining sod biomass by trial and exclosure treatment for all four sod trials. Exclosure treatments showed a pattern of greater biomass removal in the treatments accessible to lagomorphs (MEDIUM and TOTAL ACCESS) relative to other exclosure types during the pre‐monsoon trials. Following the onset of monsoons, differences in remaining biomass among treatment types disappeared. Boxplots represent the median (midline) and the first and third quartiles. Whiskers represent data points falling within 1.5 IQRs, and outliers are values >1.5 IQRs. Letters represent Tukey HSD groupings.

For the amount of remaining biomass in the sod trials, we found significant differences for both the treatments (*F*_4_,_45_ = 14.38, *p < *0.001) and the trial periods (*F*_3_,_121_ = 31.11, *p < *0.001), as well as a significant interaction between treatment and trial period (*F*_12,121_ = 6.65, *p* < 0.001; Figure 4). In both of the pre‐monsoon trials (June and early July), we found a reduction in biomass in the MEDIUM and the TOTAL ACCESS treatments (*F*_4_,_40_ = 24.54, *p < *0.001), but we did not detect a difference in treatment responses across the two pre‐monsoon trial periods (*F*_1,45_ = 1.1, *p* = 0.298). The greatest differences in remaining biomass were among the TOTAL ACCESS treatments with 29% of relative biomass remaining in June and 19% in early July (*p < *0.001 for both trial periods; all remaining biomass averages are relative to the TOTAL EXCLOSURE standardized value of 100%). Remaining biomass in the MEDIUM treatments also differed from the TOTAL EXCLOSURE treatments in the June and early July trials (*p < *0.001 for both trials) with only 44% of relative biomass remaining in June and only 29% remaining in early July. The LARGE treatments retained the majority of their relative biomass in both June (98%) and early July (80%) and did not differ from the TOTAL EXCLOSURE treatments. In the June trial, remaining biomass in the SMALL treatments did not differ from the TOTAL EXCLOSURE treatment, retaining an average of 82% of the sod biomass within this exclosure type. In our early July trial, the SMALL treatments retained an average of 68% of their relative biomass, which did not differ from the TOTAL EXCLOSURE treatment, but did differ from the TOTAL ACCESS treatments (*t = *3.383, *p = *0.012), which retained an average of only 19% of their relative biomass.

The differences in remaining biomass between the pre ($\overset{¯}{x}$ = 62.9%) and post‐monsoon ($\overset{¯}{x}$ = 100%) trial periods (*F*_1,40_ = 87.61, *p < *0.001) were driven by the differences in remaining biomass among exclosure types within the pre‐monsoon trials, which ceased completely following the onset of monsoons (late July and August trials).

From our trail camera data, we tallied a total of 2,667 herbivore detections and 35 granivore detections over the four 24‐hr sod trial periods (Table 3). Desert cottontail detections during the pre‐monsoon trials accounted for 2,077 of the 2,667 (77.9%) herbivore detections collected during the study. The number of desert cottontail detections, which averaged 1,038.5 detections per 24‐hr pre‐monsoon trial, did not differ between pre‐monsoon trial periods (June and early July) ($\chi_{1}^{2}$ = 0.139, *p = *0.7). However, only 174 desert cottontail detections were collected in the post‐monsoon trials, which was significantly fewer than the number of pre‐monsoon detections ($\chi_{1}^{2}$ = 1,608, *p < *0.001). Similarly, detections decreased in the post‐monsoon trials for both black‐tailed jackrabbits (319 pre‐monsoon, 3 post‐monsoon; $\chi_{1}^{2}$ = 310.1, *p < *0.001) and white‐throated woodrats (74 pre‐monsoon, 5 post‐monsoon; $\chi_{1}^{2}$ = 60.26, *p < *0.001). Conversely, mule deer detections increased during the post‐monsoon trials from zero detections in the pre‐monsoon trials to 15 detections in the post‐monsoon trials ($\chi_{1}^{2}$ = 15, *p < *0.001). Kangaroo rats were the only granivore that we detected during sod trials and were only detected during the early July trial (pre‐monsoon) and the late July trial (post‐monsoon). Kangaroo rat detections differed between the early July (*n = *32) and the late July (*n = *3) trials ($\chi_{1}^{2}$ = 24.02, *p < *0.001). However, the robust correlation between desert cottontail detections and biomass removal across all four trials (*R* ^2^ = 0.20, *p* < 0.001) suggests that cottontail herbivory was the primary driver of sod biomass reduction.

**Table 3:** Summary of all herbivore and granivore detections from each sod trial. Detection Count represents the total raw detections from each 24‐hr sod trial. Herbivore detections were tallied from photographs where animals were physically inside of the exclosure, whereas granivore detections were tallied from photos of animals both inside and around the exclosures during sod trials. Dietary functional groups are identified by ^H^(herbivore) and ^G^(granivore)

Species	Detection count	Percent of total detections	TOTAL ACCESS	SMALL	MEDIUM	LARGE	TOTAL EXCLOSURE
JUNE TRIAL (pre‐monsoon)							
^H^Desert cottontail	1,030	82.3	489	268^a^	273	0	0
^H^Black‐tailed jackrabbit	172	13.7	166	0	6	0	0
^H^White‐throated woodrat	49	3.9	20	17	12	0	0
^H^Mule deer	0	0	0	0	0	0	0
^G^Kangaroo rat	0	0	0	0	0	0	0
^G^“Unidentified small rodent”	0	0	0	0	0	0	0
EARLY JULY TRIAL (pre‐monsoon)							
^H^Desert cottontail	1,047	83.7	357	531^a^	159	0	0
^H^Black‐tailed jackrabbit	147	11.8	137	0	10	0	0
^H^White‐throated woodrat	25	2.0	23	0	2	0	0
^H^Mule deer	0	0	0	0	0	0	0
^G^Kangaroo rat	32	2.6	0	3	1	0	28^b^
^G^“Unidentified small rodent”	0	0	0	0	0	0	0
LATE JULY TRIAL (post‐monsoon)							
^H^Desert cottontail	149	94.9	80	0	69	0	0
^H^Black‐tailed jackrabbit	2	1.3	2	0	0	0	0
^H^White‐throated woodrat	3	1.9	3	0	0	0	0
^H^Mule deer	0	0	0	0	0	0	0
^G^Kangaroo rat	3	1.9	0	3	0	0	0
^G^“Unidentified small rodent”	0	0	0	0	0	0	0
AUGUST TRIAL (post‐monsoon)							
^H^Desert cottontail	25	58.1	10	0	15	0	0
^H^Black‐tailed jackrabbit	1	2.3	0	0	1	0	0
^H^White‐throated woodrat	2	4.7	2	0	0	0	0
^H^Mule deer	15	34.9	15	0	0	0	0
^G^Kangaroo rat	0	0	0	0	0	0	0
^G^“Unidentified small rodent”	0	0	0	0	0	0	0

a These values include detections of desert cottontails visiting the SMALL exclosures, which were designed to prevent lagomorphs from entering. While the biomass data were discarded for the SMALL exclosures that were breached by desert cottontails, the detections were retained, as they provided relevant herbivory behavior data.

b Small mammal detections were tallied by counting all detections in and around each exclosure during each 24‐hr trial period, whereas other herbivore detections were tallied by only counting detections within the exclosures.

The live‐trapping captures included a total of 62 small mammals. The majority were kangaroo rats (30.6%), followed by *Peromyscus *sp. (24.2%), and white‐throated woodrats (17.7%). Of the small mammal species captured, six are primarily granivorous and either consume green plant material in small quantities or not at all (see Table S1).

## 4 DISCUSSION

The combined seed addition trials, sod trials, and camera results show a strong association between lagomorphs, especially desert cottontails, and herbaceous biomass consumption at our semiarid shrubland study site. While other small mammals decrease above ground activity levels during the hottest periods of the summer in order to maintain body temperatures and water balances (Reichman & Van De Graaff, 1973; Walsberg, 2000), desert cottontails clearly remained active throughout the dry season, as shown by our camera data. Not only were they active, but potentially breeding, as Sowls (1957) showed that desert cottontails in Arizona have an unusually long breeding season relative to other *Sylvilagus* species. Desert cottontail populations produce young from January through August, a breeding season that spans the driest and hottest part of year (Sheppard et al., 2002; Sowls, 1957). Although little is known about the dietary demands of desert cottontails, Forys (1999) studied the differences in seasonal forage demand between sexes in two other *Sylvilagus *species (*S. virginicus* and *S. spartinae*) in Florida and found females have increased nutritional needs during gestation. The southern Arizona desert cottontail appears to have critical forage demands even during the hottest summer months, potentially due to the demands of pregnancy and heat stress, despite limited forage availability until the monsoon rains begin in July or August. High forage demands during the most water‐limited period of the year may make desert cottontails one of the most influential herbivores of grasses in arid shrublands. In areas with high local populations, herbivory pressure from desert cottontails may be one of the most critical factors in limiting the recruitment and reestablishment of grasses and forbs.

While our study primarily implicates the impact of desert cottontail herbivory, black‐tailed jackrabbits were also present in substantive numbers. Black‐tailed jackrabbits can consume large amounts of grass seedlings in semiarid shrublands, as shown by McAdoo, Longland, Cluff, and Klebenow (1987) at a study site in the Great Basin in Nevada. Even in comparison with much larger herbivores (bison), Ranglack et al. (2015) demonstrated that grass consumption by black‐tailed jackrabbits in Utah can be considerable, accounting for 34.1% of grass consumption compared to 13.7% from bison. However, Daniel et al. (1993b) showed that black‐tailed jackrabbits in the Chihuahuan Desert primarily consume shrubs, which constituted 47% of their diet. In the same region (Chihuahuan Desert), Roth, Whitford, and Steinberger (2007) showed that selective herbivory from black‐tailed jackrabbits on tarbush (*Flourensia cernua*) was enough to dramatically shift shrub dominance. These results were reinforced by the continued work of Roth et al. (2009) in the Chihuahuan Desert of New Mexico, which showed that grass clipping by small rodents combined with shrub consumption by black‐tailed jackrabbits altered the structure and composition of the vegetative community and potentially reinforced shrub dominance. Though in our study we did observe black‐tailed jackrabbits visiting our exclosures (i.e., consuming sod), we could not differentiate the impact of herbivory between desert cottontails and black‐tailed jackrabbits other than through the inference of the number of desert cottontail detections (2,077), which was much higher compared to the number of black‐tailed jackrabbit detections (319) during the pre‐monsoon trials, which suggests that desert cottontail herbivory may be more important in suppressing grass establishment at this site. The apparent reliance on shrubs by black‐tailed jackrabbits suggests potential dietary partitioning with desert cottontails, though little is known about desert cottontail diet flexibility (Brown & Krausman, 2003). Future research exploring the dietary preferences of desert cottontails and the dietary overlap between desert cottontails and black‐tailed jackrabbits may help to differentiate their relative grass consumption in arid shrublands.

In Arizona, Brown and Heske (1990) demonstrated that small rodents can have strong effects on vegetation structure in arid communities through selective granivory and other research has shown that rodents also limit grass establishment through selective herbivory and tiller cutting of newly germinated grasses (Kerley & Whitford, 2009; Kerley, Whitford, & Kay, 1997; Roth et al., 2009). White‐throated woodrats were the herbivore detected most frequently in our study after the lagomorph species and were the only rodent detected in all four trial periods. White‐throated woodrats in Arizona were shown to consume green vegetation by Dial (1988), and although measurable amounts of biomass were removed from the SMALL treatments, biomass removal in the SMALL exclosures never differed from the TOTAL EXCLOSURE control and only differed from the TOTAL ACCESS controls in the early July trial. During the early July trial period, there was less biomass remaining in both the SMALL and TOTAL ACCESS exclosure treatments relative to the June trial, as well as a high number of desert cottontail detections (1,047), suggesting the differences between these two exclosure types could be attributed to a high degree of lagomorph herbivory in the TOTAL ACCESS exclosure and not biomass consumption by rodents in the SMALL exclosures. Although we had reduced detection ability for kangaroo rats and other small rodents (i.e., the “unidentified small rodent” category) relative to other herbivore size classes, the low rate of biomass removal in the SMALL cages was consistent with their relatively low rate of detection, suggesting that the influence of rodent herbivory is far less consequential than that of lagomorphs. Additionally, the majority of the small rodents present on the study site are primarily granivorous (see Table S1), which suggests the consumption of green biomass by the small rodent community as a whole is likely minimal.

The differences in biomass removal between exclosure types and the rate of herbivore detection both shifted dramatically at the onset of monsoon rains. Although Smith (1990) showed that black‐tailed jackrabbit home ranges in the western United States can range anywhere from 100 to 300 ha, well over the size of our study area, Chapman and Willner (1978) reported the home range size of desert cottontails in the same region to be much smaller, ranging from 0.4 to 6.1 ha, small enough for multiple desert cottontail home ranges to fall within the boundaries of our ~11 ha study site. Neither species is known to be migratory, and it was assumed that the same number of resident animals was present prior to and following the monsoons. Previous research by Chapman and Willner (1978) on the diets of desert cottontails and Fatehi, Pieper, and Beck (1988) on the diets of black‐tailed jackrabbits showed that the diets of both species vary seasonally and are strongly influenced by moisture availability. Because our pre‐monsoon trial periods took place during the period that is generally the driest part of the year (Sheppard et al., 2002), when water stress is most pronounced for both plants and animals, the divisions of fresh sod introduced into our exclosures represented a highly desirable food source. This is evidenced by the high degree of biomass consumption over a very short amount of time (24‐hr.) during both the June and early July trials, as well as the effort required for desert cottontails to breach our SMALL exclosures in the June and early July trials (e.g., digging and squeezing through small entrances). The desirability of green forage suggests that although the forage demand seemed to decrease following the onset of the rains, once the annual monsoon cycle ceases forage demand and herbivory pressure will once again increase and lagomorphs will consume any remaining herbaceous biomass that had germinated during the monsoons. The current conditions of the study site seem to reflect this cycle of forage demand, as numerous herbaceous plants emerged following summer rains, but very little herbaceous plant material persisted into the dry summer months. Further research on lagomorph diets that focus on seasonal dietary shifts may better establish their impact on grass establishment in semiarid shrublands.

If increasing grass cover is an important component of regional management goals, then herbivory impacts and population dynamics of native lagomorphs need to be considered by land managers when attempting shrubland‐to‐grassland restoration projects. The low recruitment from seeds observed in this experiment is a common challenge to restoration in the region, as recruitment relies on both the amount and distribution of rainfall, which can be highly variable from year to year (Fehmi, Niu, Scott, & Mathias, 2014). The compounding effects of high herbivory pressure and low recruitment due to the variability of rainfall may substantially limit and prolong the recovery of grasses following livestock removal, which can already take decades (Valone et al., 2002). Shrub presence alone can be considered to be a negative impact to grassland systems, but shrubs also act as an important landscape component for predator avoidance for black‐tailed jackrabbits (Arias‐Del Razo, Hernández, Laundré, & Velasco‐Vázquez, 2012). Similarly, desert cottontails may respond to the improved predator avoidance conferred by increased shrub cover through increased occupancy in shrub‐encroached areas, potentially creating a positive feedback loop of intensified herbivory pressure in shrub‐encroached regions, further hampering restoration efforts.

Our study suggests that by establishing herbivore exclosures that limit desert cottontail herbivory, herbaceous biomass retention can be increased, and given adequate rainfall, recruitment from seed may be improved. However, logistical and financial constraints may limit the establishment of fencing or exclosures at larger spatial scales, in which case managers might consider first thinning the shrub density to discourage the site's use by lagomorphs, thus decreasing the level of lagomorph herbivory pressure. Additionally, creating habitat to promote populations of natural lagomorph predators including the installation of raptor perches, owl boxes, and maintaining landscape complexity on a site's perimeter to allow the approach of mammalian predators (i.e., coyotes) may help control lagomorph populations and limit their impact on grasses and herbaceous plants (Banks, 2000; Banks, Dickman, & Newsome, 1998; Henke & Bryant, 1999).

## CONFLICT OF INTEREST

None declared.

## AUTHOR'S CONTRIBUTIONS

All authors contributed to the research design. S.A. collected the data and wrote the manuscript. M.N. secured site permissions. S.A., J.F., and J.K. analyzed the data. J.F., J.K., and M.N. provided edits. All authors provided approval for publication.

## DATA ACCESSIBILITY STATEMENT

The raw data relevant to all analyses in this manuscript were archived with Dryad under https://doi.org/10.5061/dryad.0f8p4c5.
