# Supplementary material for: Native lagomorphs suppress grass establishment in a shrub‐encroached, semiarid grassland
Source: Ecol Evol. 2018 Dec 18;9(1):307–17. doi: 10.1002/ece3.4730 (PMC6342093; doi:10.1002/ece3.4730)
Supplement: Supplementary file 1 [file ECE3-9-307-s001.docx]

**Table S1.** Summary of dietary preferences of herbivore species known to occupy the study site via camera-trapping and live-trapping. The majority of the rodents at the study site are primarily granivorous. Other species, such as mule deer, desert cottontails, and black-tailed jackrabbits, feed opportunistically based on seasonal forage availability. Detection method in Total Number of Detection column is distinguished by ^L^ (live-trapping) and ^C^ (camera-trapping).

| Common Name | Species | Total Number of Detections | Primary Dietary Preferences | Secondary Dietary Preferences | Citation |  |
| --- | --- | --- | --- | --- | --- | --- |
| Bailey’s pocket mouse | *Chaetodipus baileyi* | 2^L^ | Seeds | Green plant material, insects | Paulson (1988) | |
| Desert pocket mouse | *Chaetodipus penicillatus* | 11 ^L^ | Seeds | Insects | Mantooth and Best (2005) | |
| Merriam’s kangaroo rat | *Dipodomys merriami* | 19 ^L^ /776 ^C^ | Seeds | Green plant material, insects | Smigel and Rosenzweig (1974); Kerley, Whitford and Kay (1997); Reid (2006) | |
| Ord’s kangaroo rat | *Dipodomys ordii* | 1 ^L^ | Seeds | Green plant material, insects | Garrison and Best (1990) | |
| White-throated woodrat | *Neotoma albigula* | 11 ^L^ /3,945 ^C^ | Forbs and woody plant material | Grasses | Dial (1988) | |
| Mule deer | *Odocoileus hemionus* | 7,210 ^C^ | Shrub and tree browse | Grass, forbs | Krausman *et al.* (1997) | |
| Southern grasshopper mouse | *Onychomys torridus* | 3 ^L^ | Invertebrates | Reptiles, amphibians | McCarty (1975) | |
| n/a | *Peromyscus* sp. | 11 ^L^ |  |  |  | |
| Silky pocket mouse | *Perognathus flavus* | 3 ^L^ | Seeds |  | Best and Skupski (1994) | |
| Cactus mouse | *Peromyscus eremicus* | 1 ^L^ | Seeds, invertebrates | Green plant material | Veal and Caire (1979) | |
| Rock squirrel | *Spermophilus variegatus* | 11 ^C^ | Seasonally available plant material | Nuts, seeds, grain, berries, fruit, roots, green vegetation, cactus, invertebrates, fresh and dried meat | Oaks, Young, Kirkland and Schmidt (1987) | |
| Desert cottontail | *Sylvilagus audubonii* | 38,357 ^C^ | Seasonally available plant material | Grasses, sedges, rushes, herbs, forbs, tree saplings | Chapman and Willner (1978) | |
| Black-tailed jackrabbit | *Lepus californicus* | 7,869^C^ | Seasonally available plant material | Grasses forbs, shrubs | Vorhies and Taylor (1933); Fatehi, Pieper and Beck (1988) | |
